# Novel Post-Harvest Preservation Techniques for Edible Fungi: A Review

**DOI:** 10.3390/foods13101554

**Published:** 2024-05-16

**Authors:** Yuping Cao, Li Wu, Qing Xia, Kexin Yi, Yibin Li

**Affiliations:** 1 College of Food Science, Fujian Agriculture and Forestry University, Fuzhou 350002, China; caoyp12@163.com (Y.C.); xiaqing_830@163.com (Q.X.); kexiny09@163.com (K.Y.); 2 Institute of Food Science and Technology, Fujian Academy of Agricultural Sciences, Fuzhou 350003, China; xxj1963@163.com; 3 National R&D Center for Edible Fungi Processing, Fuzhou 350003, China; 4 Key Laboratory of Subtropical Characteristic Fruits, Vegetables and Edible Fungi Processing (Co-Construction by Ministry and Province), Ministry of Agriculture and Rural Affairs, Fuzhou 350003, China; 5 Fujian Province Key Laboratory of Agricultural Products (Food) Processing Technology, Fuzhou 350003, China

**Keywords:** edible fungi, new technologies, post-harvest preservation, nutraceutical value

## Abstract

Edible fungi are well known for their rich nutrition and unique flavor. However, their post-harvest shelf-life is relatively short, and effective post-harvest preservation techniques are crucial for maintaining their quality. In recent years, many new technologies have been used for the preservation of edible fungi. These technologies include cold plasma treatment, electrostatic field treatment, active packaging, edible coatings, antimicrobial photodynamic therapy, and genetic editing, among others. This paper reviews the new methods for post-harvest preservation of mainstream edible fungi. By comprehensively evaluating the relative advantages and limitations of these new technologies, their potential and challenges in practical applications are inferred. The paper also proposes directions and suggestions for the future development of edible fungi preservation, aiming to provide reference and guidance for improving the quality of edible fungi products and extending their shelf-life.

## 1. Introduction

Edible fungi, commonly known as mushrooms, refer to a type of fungi that are safe for human consumption. They are rich in variety, with common mainstream edible fungi including *Agaricus bisporus*, *Lentinus edodes*, *Flammulina velutipes*, *Pleurotus ostreatus*, *Tremella fuciformis*, etc. Edible fungi have high nutritional value and contain high-quality proteins, dietary fiber, vitamins, and minerals. They are also rich sources of bioactive substances such as polysaccharides, polyphenols, terpenoids, etc. [1]. The presence of various beneficial components not only enriches the nutritional content of mushrooms but also gives them medicinal properties. For example, *T. fuciformis* contains a polysaccharide content of up to 60~70% [2]. This component has been widely extracted and researched, demonstrating preventive effects on various diseases such as cancer, cardiovascular diseases, and diabetes [3,4]. Nowadays, people pursue healthy dietary and lifestyle choices. Edible fungi are low in fat and have a protein content higher than most vegetables [5]. They are also the only non-animal food that provides a significant amount of vitamin D_2_ [6]. The vitamin D_2_ content in every 100 g of fresh edible fungi is equivalent to the daily requirement recommended internationally, making them an important source of vitamin D_2_ for vegetarians [7,8]. Consequently, an increasing number of consumers are incorporating edible fungi into their diets, further expanding market demand and driving the prosperity and innovation of the edible fungi industry. According to research forecasts, the global market demand is expected to reach 20.84 million tons by 2026 [9].

However, the tender texture of fresh edible fungi poses significant challenges to their commercial distribution after harvest. During harvesting, transportation, storage, and retail processes, the quality of fresh mushrooms can be affected by various factors. For example, post-harvest metabolic activity of the fungi may lead to weight loss, cap opening, and elongation of the stem. Mechanical damage or invasion by various pathogenic microorganisms may cause a series of decay and deterioration phenomena in the fungi, such as browning, softening, and the emission of unpleasant odors [10,11]. All of these phenomena can significantly reduce the commercial and culinary value of edible fungi. Therefore, the utilization of suitable and efficient post-harvest preservation methods to prolong the storage period of edible fungi and enhance their economic value has been a hot topic of concern.

In recent years, numerous new technologies have emerged in the field of edible fungi preservation. These include applications such as cold plasma treatment [12], electrostatic field treatment [13], active packaging [14], edible coatings [15], antimicrobial photodynamic therapy [16], electrolyzed water treatment [17], novel preservatives [18], and genetic editing [19]. They demonstrate significant advantages in the preservation of edible fungi. For instance, antimicrobial photodynamic therapy can more precisely target microorganisms, reducing the impact on food itself and environmental pollution compared to traditional heat and chemical sterilization mechanisms [20]. Edible coatings and electrolyzed water treatment align with the concept of sustainability [21,22]; electrostatic field treatment serves as a reliable auxiliary means for refrigeration preservation [23]; the application of active packaging and smart packaging improves the stability of preservation effects [24]; the use of novel preservatives further improves food safety [25]; genetic editing is advanced biotechnology that updates people’s understanding of post-harvest preservation of edible fungi [26]. These innovative technologies play an important role in improving the overall quality and sustainability of food, injecting new vitality into the food industry.

## 2. Significance of Post-Harvest Preservation of Edible Fungi 

**Figure 1:**
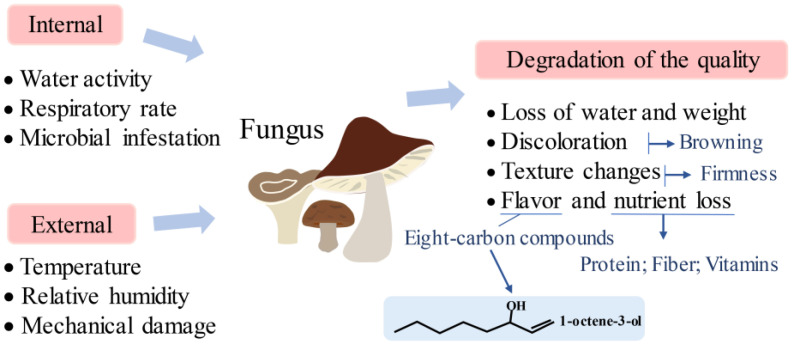
Post-harvest quality degradation of edible fungi and its influencing factors.

Fresh edible fungi are highly perishable food items, with a shelf-life of only 1~3 days at room temperature and 5~7 days under refrigeration conditions [27]. This implies that effective preservation methods are crucial for maintaining the post-harvest quality of edible fungi. Figure 1 illustrates the vulnerability of post-harvest edible fungi.

The deterioration of its quality is mainly manifested by moisture loss, weight reduction, softening texture, discoloration, and a decrease in flavor compounds and nutritional content [1]. Many internal and external factors, such as the water activity of edible fungi, respiration rate, microbial activity, relative humidity, temperature, and mechanical damage, all influence the deterioration of its quality [28]. Exploring the mechanisms underlying the decline in its post-harvest quality contributes to the innovative development of preservation technologies.

Fresh mushrooms have a water content of approximately 90%, hence their tender and juicy texture [10]. However, due to the lack of protective tissues against microbial attacks and moisture loss on the surface, as well as the influence of transpiration, if protective measures are not taken immediately after harvesting, a large amount of moisture will be lost, resulting in tissue shrinkage and weight loss [11]. During storage, respiratory and other life activities consume nutrients in the fruiting bodies. This loss of nutrients also leads to weight loss [29]. Typical measures include cooling [30] and packaging [31] to slow down the loss of moisture and nutrients.

With prolonged post-harvest time, the texture and color of mushrooms undergo changes. This is mainly associated with the activity of a series of enzymes. Enzymes such as cellulases, chitinases, and β-1,3-glucanases degrade components of the tissue cell wall, resulting in the softening of mushrooms [32,33]. Enhanced activities of enzymes such as phenylalanine ammonia-lyase, cinnamyl alcohol dehydrogenase, and peroxidase can lead to the accumulation of lignin [34]. The accumulation of lignin causes the mushroom tissue structure to become rough, resulting in a decrease in palatability. Polyphenol oxidase catalyzes enzyme-catalyzed reactions, forming a large amount of dark-colored substances that cause discoloration of mushrooms [35]. Some pathogens, especially *Pseudomonas*, have a destructive effect on the mushroom cell membrane and can participate in the activation process of phenol oxidase. They trigger enzyme-catalyzed browning by promoting the reaction between phenol oxidase and intracellular substrates bidirectionally [36]. Mechanical damage causes the leakage of cell contents, promoting the contact reaction between substrates and phenol. Temperature fluctuations have a significant impact on enzyme activity. High humidity accelerates the growth of harmful microorganisms [37]. Intense respiration promotes browning reactions. Additionally, research has indicated that a decrease in protein content also leads to softening of mushrooms [32]. Oxidation reactions such as non-enzyme-catalyzed browning directly result in the darkening of mushroom color [38].

Post-harvest microbial decay of edible mushrooms is a critical issue because they may become contaminated by bacteria, fungi, or other microorganisms during planting, harvesting, processing, and storage [39,40,41]. *Pseudomonas*, *Enterobacter*, *Erwinia*, *Pantoea*, and *Rahnella* are the main spoilage bacteria in edible mushrooms [42,43,44,45]. *Verticillium*, *Cladobotryum*, and *M. perniciosa* are the main decay fungi, along with other microorganisms such as dsRNA viruses and ssRNA viruses [39]. They decompose mushroom components by secreting various enzymes, competitively consume nutrients such as proteins and carbohydrates, produce toxins, promote the formation of decay conditions, and ultimately lead to the softening and decay of fruiting bodies [46,47,48]. Among them, *Pseudomonas* is one of the important microorganisms causing post-harvest spoilage of edible mushrooms [47]. For example, an increase in the relative abundance of *Pseudomonadaceae* was observed in *P. ostreatus*, leading to spoilage [45]. The pathogen primarily responsible for the epidemic bacterial blotch disease in *A. bisporus* was *Pseudomonas* [49]. *Pseudomonas* also induced apoptosis of cells in *F. velutipes*, hydrolyzed proteins, and polysaccharides, resulting in slow mycelial growth and significant yield losses [50]. Other pathogens may also lead to microbial spoilage of edible mushrooms post-harvest. For example, *Burkholderia gladioli* pv. Agaricicola could cause hollow disease in *A. bisporus* [51]. Additionally, fungi such as *Cystofilobasidium*, *Aspergillus*, and *Mucor* have a significant impact on the post-harvest quality of wild morel mushrooms [43]. Therefore, microbial control after the harvest of edible fungus holds significant importance.

The unique aroma and umami taste of mushrooms are essential characteristics. The presence of volatile compounds such as C-8 compounds imparts key aroma characteristics to mushrooms [52]. The umami taste is mainly attributed to umami amino acids and 5′-nucleotides [53]. According to current research on the post-harvest changes in the umami taste and aroma of mushrooms, it is found that they are mainly related to nucleotide metabolism, amino acid metabolism, fatty acid metabolism, and other metabolic pathways [54]. For instance, one study conducted comprehensive physiological and transcriptomic analyses, revealing that a high-energy state helped maintain the umami taste of mushrooms [55].

## 3. Emerging Preservation Technologies

Traditional post-harvest preservation techniques for edible fungi have certain limitations [56]. For example, irradiation preservation and excessive heat treatment may lead to the loss of food nutrients [11,57,58]; the use of chemical disinfectants has adverse effects on human health and the environment [25]; traditional packaging materials used in modified atmosphere packaging are non-biodegradable [17]. In contrast, emerging preservation technologies such as cold plasma treatment [12], electrostatic field treatment [13], active packaging [14], edible coatings [15], antimicrobial photodynamic therapy [16], electrolyzed water treatment [22], novel preservatives [18], and genetic editing [19] not only effectively extend the shelf-life of food but also focus on preserving their sensory characteristics and nutritional value, while adhering to the concept of sustainable development. The emergence of these new technologies provides safer and more efficient preservation solutions, bringing new hope and opportunities to the food industry. Next, a brief review of emerging preservation technologies in recent years will be provided.

### 3.1. Packaging

Currently, common packaging materials used for preservation mainly include polyethylene, polyvinyl chloride, and polypropylene. However, these materials have low permeability and moisture permeability, which will lead to excessive accumulation of CO_2_ and condensation of water vapor on the film surface. Compared to the aforementioned packaging materials, the use of micro-perforated film improves permeability. In one study, microporous membranes maintained the ideal color of *A. bisporus* by inhibiting the formation of condensation water and harmful volatile compounds inside the membrane [59]. In another study, microporous membrane packaging reduced the generation of odor compounds, thus positively affecting flavor retention and extending the preservation of *A. bisporus* [60]. With the continuous improvement in the requirements for packaging materials, multifunctional nanocomposite materials with better mechanical properties and preservation effects have become a research hotspot in the packaging field [61]. One research team prepared polyethylene-based packaging materials loaded with nano-Ag and nano-TiO_2_ and found that nanoparticles alleviated cell membrane damage in *A. bisporus* by affecting membrane lipid metabolism processes [62]. Another research team explored the mechanism of nanocomposite packaging materials in inhibiting mushroom browning. They found that nanocomposite packaging materials could maintain the total phenol content and inhibit the activities of various enzymes (such as polyphenol oxidase) and related gene expression pathways involved in melanin formation, thus reducing melanin formation and delaying browning of *A. bisporus* [63].

Active packaging is an innovative packaging system containing active ingredients [64]. It can exert antimicrobial, moisture-resistant, antioxidant, and odor-resistant effects on packaged food by releasing active agents. Electrospinning technology is a versatile technique for designing active packaging [65]. Biologically active paper loaded with 1-methylcyclopropene (1-MCP) can delay the softening, browning, and weight loss of *L. edodes* by adsorbing and removing ethylene inside and outside the packaging [66]. MgO nanoparticles and grape seed oil were loaded into poly(3-hydroxybutyrate) thin films, and it was found that the antibacterial and antioxidant activities of the films were enhanced, and the growth of *Staphylococcus aureus* and *Escherichia coli* was inhibited, thereby extending the shelf-life of *A. bisporus* to 6 days under room temperature storage conditions [67]. The control of the release amount and rate of active substances in packaging is a focus of later-stage research [68].

Intelligent packaging is an advanced packaging technology with integrated sensors and monitoring devices that enable tracking, monitoring, and managing packaged products [69,70]. Intelligent packaging mainly comes in two application forms: smart controlled release and smart response [71]. From the perspective of intelligent controlled release, intelligent packaging releases active substances by sensing environmental stimuli to mitigate the adverse effects of environmental changes on food products. For example, hydrogel-controlled release packaging was able to regulate the release of 1-MCP to inhibit ethylene-induced aging processes [72]. A hybrid aerogel prepared using pectin and cellulose nanofibers stabilized humidity within the membrane by controlling catechol release, thereby delaying the quality deterioration of *A. bisporus* [73]. Intelligent, responsive packaging can monitor environmental conditions and product status in real time and provide feedback to consumers through various interactive means. The application of intelligent packaging technology is pushing the preservation of edible mushrooms in more intelligent and sustainable directions, making it an outstanding innovation in the packaging field today. Some emerging packaging films for edible fungi are shown in Table 1.

### 3.2. Cold Plasma Treatment

**Figure 2:**
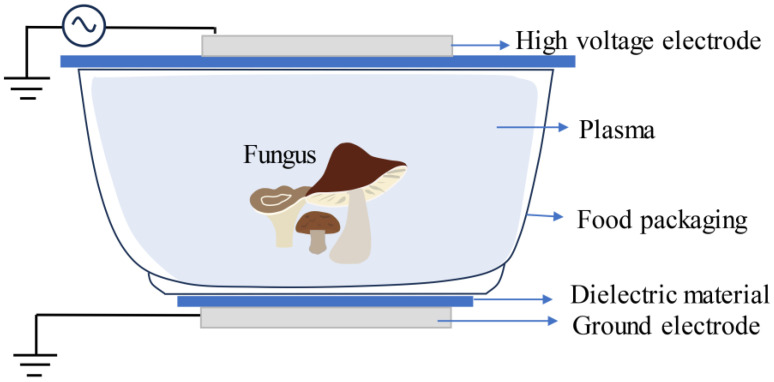
Dielectric barrier discharge (DBD) treatment.

The food industry is actively seeking new non-thermal food processing technologies [78]. In recent years, cold plasma (CP) treatment has attracted considerable attention as a novel cold sterilization and preservation technology [79]. Plasma is the fourth state of matter in nature, generated by the decomposition of air by high-energy electrons [78,80]. In the preservation of edible mushrooms, dielectric barrier discharge (DBD) is the most effective method for producing CP [81]. The key lies in sealing the product and gas inside the packaging, generating a strong electric field under external electrode action, ionizing the gas inside the packaging, and forming sterilizing plasma (Figure 2) [82]. In a study, when 30% hydrogen peroxide steam (flow rate of 0.47 mL/min) and argon (4.24 L/min) were used as working gases, DBD treatment prolonged the storage period of *A. bisporus* by inhibiting enzymatic browning and inactivating *Pseudomonas* [12]. In another study, when air was used as the working gas, DBD treatment effectively inhibited microbial growth and reproduction while reducing browning reactions and oxidative damage, thus maintaining the color and texture of *F. velutipes* [83]. A research team conducted a comparative analysis of the effects of DBD treatment and direct cold plasma treatment on the physicochemical properties and shelf-life of *A. bisporus* [84]. The results showed that DBD treatment was more effective in inhibiting the total number of bacteria, yeast, and mold while also resulting in lower browning value and better quality characteristics of the mushrooms.

The water treated by CP is called Plasma-Activated Water (PAW) [85]. After soaking in PAW, *A. bisporus* deactivates *E.coli* on its surface, delaying the softening and browning process [86]. Previous research [87] compared and analyzed the preservation effects on mushrooms with four different treatment groups: the DBD treatment group; the PAW treatment group; the pure water treatment group; and the control group. The results showed that the mushrooms in the PAW treatment group had the lowest browning index and the best hardness and sensory performance. It may be because PAW treatment increases contact with the uneven surface of mushrooms, and compared to direct plasma treatment, the main active components of PAW are reactive oxygen and nitrogen, which are more targeted at killing pathogenic microorganisms [88]. PAW treatment is an optimization and improvement of CP preservation technology. However, soaking mushrooms in water for washing may cause mechanical damage to tissues and water absorption. It is worth considering whether PAW treatment will affect the texture of mushroom fruiting bodies. In the future, further exploration should be conducted to determine the optimal application conditions of CP technology in the preservation of edible mushrooms, providing a more reliable scientific basis for its application.

### 3.3. Edible Coating

Edible coatings have long been of great interest due to their edibility and sustainability. Edible coatings are thin layers formed by directly immersing or spraying food-grade coatings onto the surface of food and drying them [21]. Most edible coating substrates, such as alginate [89], cellulose [90], chitosan [91], gelatin [92], plant proteins [93], and phospholipids [94], are derived from natural animals and plants to develop effective edible coating materials for mushrooms. Essential oils, flavonoids, and other active ingredients are integrated into edible coatings. The addition of these substances enhances the antioxidant, antibacterial, and anti-pathogenic microorganism properties of the coatings. Additionally, edible coatings can improve the utilization rate of active ingredients through sustained release and avoid the adverse effects of unstable volatilization on the flavor of edible mushrooms [95]. Some recent coatings are shown in Table 2.

Natural plant essential oils possess potent antioxidant and antibacterial properties, making them typical bioactive substances for enhancing packaging performance [96,97]. The effect of incorporating cinnamaldehyde essential oil nanoemulsion (CIN) into alginate-based edible coatings on mushroom preservation was studied. The results revealed that the addition of plant essential oil CIN reduced the respiration rate, weight loss, and the number of pathogenic bacteria such as *Pseudomonas* in *A. bisporus*, thereby enhancing antioxidant capacity and improving the preservative properties of the composite coating [15]. An edible coating prepared with aloe vera gel loaded with orange peel essential oils extended the shelf-life of button mushrooms after harvest to 16 days [98]. Developing edible coatings represents a significant step for the packaging industry toward a healthier and more sustainable direction.

**Table 2:** Recent edible coatings.

Mushroom Species	Packaging Materials	Best Rations	Result	Ref.
*A. bisporus*	Cellulose nanocrystals (CNCs)/gellan gum	____	The input and output of gases are controlled; the respiration rate is suppressed	2021 [29]
*A. bisporus*	Cinnamaldehyde (CIN)/ alginate/Tween 80	Oil: emulsifier (1:1); 0.05 mL/100 mL CIN	Decreased respiration rate and *Pseudomonas* counts; increased antioxidant and firmness retention.	2021 [15]
*A. bisporus*	Protocatechuic acid (PA)/CaCl_2_/NaCl/pullulan (Pul)	118 mg/L PA; 0.83% CaCl_2_; 0.55% NaCl; 0.30% Pul	Suppressed respiration rate, browning, and flavor loss; increased antioxidant activity; prolonged shelf-life to 16 days	2022 [99]
*A. bisporus*	*Salvia macrosiphon* seed (SSG)/liquid smoke (LS)	3% LS	Delayed weight loss, softening, and browning; enhanced total phenolic content	2023 [100]
*A. bisporus*	Aloe vera gel/orange peel essential oil (EOs)	1500 µL/L Eos; 50% aloe vera gel	Suppressed respiration rate; prolonged shelf-life to 16 days; enhanced antioxidant activity	2023 [98]
*A. bisporus*	Glycerol/citric acid/polysaccharides aqueous extracts from *P. eryngii*	____	Inhibited dehydration and degradation; delayed browning	2023 [101]
*A. bisporus*	Chia seed mucilage/*Ferula gummosa* (FG) and *Ziziphora clinopodioide*s (ZC) essential oils	500 ppm ZC	Reduced weight loss, browning; enhanced firmness feature; extended the shelf-life up to 16 days	2024 [102]
*A. bisporus*	Guar gum/leek powder (LP) /sunflower oil (SO)	1.5% LP; 0% SO	Preserved the moisture, shape, and color quality	2023 [103]
*L. edodes*	γ-polyglutamic acid hydrogel	1%	Inhibited water and weight loss, decay, and Vitamin C degradation; reduced polyphenol oxidase activity	2021 [104]
*L. edodes*	Polysaccharide from *Oudemansiella radicata*	____	Improved retention of nutritional and flavor compounds; delayed softening; reduced MDA production	2021 [105]
*F. velutipes*	Pullulan (Pul)/cinnamaldehyde (CA)/soybean phospholipids (SP)	6% Pul	Delayed color change; increased antioxidant activity	2023 [106]

Note: “____” indicates that there is no best rations.

### 3.4. Antimicrobial Photodynamic Therapy

Antimicrobial photodynamic therapy (APDT) is an innovative food sterilization technique [107]. It works by irradiating a light source to activate a photosensitizer, generating reactive oxygen species such as singlet oxygen and free radicals, thereby achieving the eradication of bacteria, fungi, parasites, and other microorganisms in food [107]. Photosensitizers are typically colored compounds that absorb light at specific wavelengths, such as curcumin and riboflavin [107,108]. Compared to traditional heat treatment and chemical sterilization methods, APDT is gentle, residue-free, and does not lead to the development of microbial resistance in pathogens. For instance, curcumin-mediated APDT successfully reduced the bacterial count on the surface of *T. fuciformis* and retained the color, moisture content, and hardness [16]. Many studies have combined APDT with composite films for food preservation [109,110]. For example, curcumin was used as a photosensitizer to prepare chitosan-based films loaded with silver nanoparticles [111] and konjac glucomannan-based antibacterial films [112]. The addition of natural photosensitizers enhanced the mechanical properties, antibacterial performance, and antioxidant activity of the films. The film packaging reinforced the stability of the photosensitizer, and its excellent barrier properties effectively prevented secondary infection after APDT. However, the penetration power of the light source in APDT is limited, posing a significant challenge in eradicating microorganisms hidden in the gills of edible mushrooms.

### 3.5. Electrostatic Field Treatment

Electrostatic field treatment is a non-thermal physical preservation technique that is typically used as an adjunct to refrigeration to extend the shelf-life [113]. It works by ionizing the air to create a negative ion environment, thereby inhibiting the metabolism of fruits and vegetables, suppressing the growth of surface microorganisms, and affecting enzyme activity simultaneously [114]. Electrostatic field treatment is classified into high-voltage electrostatic field (HVEF) treatment (>2.5 kV) and low-voltage electrostatic field (LVEF) treatment (≤2.5 kV) based on the output voltage [113]. It does not cause significant changes in food temperature during the treatment process, making it suitable for heat-sensitive foods such as mushrooms [23]. Research has shown that treating *A. bisporus* with HVEF can reduce hardness loss, enhance antioxidant enzyme activity, and induce the breakdown of oxidative enzymes [115]. In other research, Liu combined LVEF with modified atmosphere packaging (MAP) to investigate its effect on the post-harvest shelf-life of *A. bisporus*. The results showed that compared to the sole use of MAP treatment, the use of LVEF reduced the respiratory rate of mushrooms, inhibited the proliferation of pathogenic microorganisms, and extended the shelf-life of mushrooms from 6 days to 12 days [13].

The HVEF preservation technology using DENBA+ electrostatic device is referred to as “DENBA+ technology”. Its preservation principle lies in installing DENBA+ electrode plates in the refrigerated space, utilizing high-voltage electrostatics to generate electromagnetic static waves. These waves resonate and activate water molecules in food, disturbing the internal metabolic processes of food cells and thereby slowing down food decay [116]. DENBA+ technology holds promising prospects in the field of food preservation and has already begun commercialization. In a study aimed at extending the shelf-life of strawberries with DENBA+-assisted refrigeration, it was found that DENBA+ technology could inhibit the respiration rate and substance metabolism of strawberries, delay the decline in texture and soluble solids content, kill pathogenic bacteria, reduce their decay index, thus extending the shelf-life [117]. Compared to other application forms and devices in high-voltage electrostatic field treatment, the advantages of DENBA+ technology lie in emitting uniformly distributed beam-like static electricity, which expands the electric field. Increasing the electric field strength and achieving uniform electric field density is advantageous for preservation treatment. Additionally, DENBA+ technology is energy-saving and environmentally friendly, with simple device setup and convenient installation. This technology has demonstrated promising results in preserving fruits and vegetables. Thus, it is worthwhile to explore its application further in mushroom preservation.

### 3.6. Electrolyzed Water

Electrolyzed water (EW) is water containing active oxygen substances produced by the electrolysis of neutral salt solutions, possessing excellent disinfection, bacteriostatic, and cleaning functions. It mainly destroys microbial cells and internal structures, affecting the growth of harmful microorganisms on the surface of edible mushrooms by generating active oxygen substances and adjusting the acidity and alkalinity of the environment [22]. The effective chlorine concentration (ACC) and oxidation-reduction potential (ORP) determine the antibacterial activity of EW [118]. Research has explored the mechanism of slightly acidic electrolyzed water (SAEW) in inhibiting the activity of mushroom polyphenol oxidase. One study found that the HOCl component in SAEW can not only reversibly bind to polyphenol oxidase, hindering the catalytic action between the enzyme and the substrate, but also inhibit the formation of many compounds related to melanin, thereby delaying the browning process of mushrooms [119]. The browning index of *A. bisporus* treated with 25 mg/L electrolyzed water was lower than that of untreated mushrooms [22]. In another study, the bactericidal efficacy of electrolyzed water was compared with several other sterilizers. It was found that under room temperature conditions (23 ± 2 °C), electrolyzed water had the strongest effect on foodborne pathogens in *P. ostreatus*, reducing the total aerobic bacterial count, total mold count, and the number of pathogenic bacteria by 1.35 log CFU/g, 1.08 log CFU/g, and 1.90~2.16 log CFU/g, respectively, with significant bactericidal effects [120].

Electrolyzed water has strong antibacterial activity, leaves no residue, and is easy to produce, making it a broad-spectrum bacteriostatic agent with promising prospects. However, immersing edible mushrooms in water for washing may cause mechanical damage to tissues and water absorption. Moreover, microorganisms may develop resistance to the active ingredients in electrolyzed water, reducing its bactericidal effectiveness. Therefore, the lifespan of electrolyzed water is short, requiring frequent replacement and resulting in high usage costs. In the future, it is necessary to establish and improve relevant technologies to promote the development and application of electrolyzed water in the preservation of edible mushrooms.

### 3.7. Novel Preservatives

The safety of food preservatives is a significant concern. For instance, the use of traditional preservatives like sodium hypochlorite may pose health risks [121]. Extracts and secondary metabolites extracted from natural sources such as plants, animals, and microorganisms are becoming a trend as novel preservatives [25]. Preservatives act on food through methods such as soaking, immersing, spraying, or fumigating, exhibiting antibacterial, antioxidant, anti-browning, and anti-aging properties [18]. For example, spraying ergothioneine on the surface of *A. bisporus* maintained higher levels of total phenolics and ascorbic acid, thereby slowing down the browning process [122]. Similarly, immersing *A. bisporus* in exogenous γ-aminobutyric acid increased the activity of mushroom phenylalanine ammonia-lyase and gene expression, thereby delaying the browning process during refrigeration [123]. A 1-MCP is a common and efficient ethylene inhibitor that can irreversibly bind to ethylene receptors, thereby preventing ethylene-induced ripening and aging processes [124]. In recent years, 1-MCP treatment has been applied as a new preservation method for edible mushrooms. Studies have found that combining 1-MCP with low-permeability packaging with limited oxygen supply can significantly reduce the respiration rate of *A. bisporus* by approximately 25%, extending the shelf-life to over 15 days [125]. In another study, *P. ostreatus* treated with 1-MCP exhibited lower ethylene production peaks and higher energy charges, effectively preserving the freshness and sweetness of mushrooms [126]. Essential oils, natural aromatic oil extracts with strong volatility, exhibit excellent antioxidant and antibacterial activities, typically employed in the form of fumigation [127]. Fumigating *A. bisporus* with peppermint oil enhanced the hardness, total phenolics, and ascorbic acid content of mushrooms, reduced weight loss, and delayed the aging process of mushrooms [128]. Recent studies have found that films loaded with essential oils could effectively maintain the post-harvest quality of button mushrooms [129,130]. In the future, various antioxidants can be combined with innovative packaging materials such as films and preservation paper to promote the application of novel preservatives in the field of edible mushrooms. Furthermore, further research is needed on the potential mechanisms of various novel preservatives to enhance their safety and effectiveness.

### 3.8. Other Emerging Methods

The emergence of gene editing technology has provided novel possibilities for mushroom preservation. Research has shown that editing the PPO1 gene of *A. bisporus* using the CRISPR/Cas9 method significantly reduced the degree of browning in the edited mushrooms, providing a new strategy for extending their storage period [26]. Two hybrid ethylene receptors, AbETR1 and AbETR2, have been identified in *A. bisporus*, and by downregulating the expression of AbETR1 and AbETR2, the maturation and senescence of mushroom fruit bodies are inhibited [19]. In recent years, many researchers have conducted editing, decoding, and sequencing of mushroom genomes, laying the foundation for the biological and genetic research of mushrooms [131,132].

Furthermore, research has found that ultrasound treatment may have a potential impact on maintaining mitochondrial energy supply in mushrooms [133]. In a study, Shi combined treatment of ultrasound and irradiation reduced the adhesion of microorganisms such as *Pseudomonas aeruginosa* and *Enterobacteriaceae*, alleviating browning and moisture loss in fresh mushrooms [134]. Air-ion treatment has a positive effect on maintaining the energy and flavor of fresh *L. edodes*, controlling browning and post-harvest quality loss [135]. Pulse light and pulsed electric fields are also effective choices for inactivating harmful microorganisms and controlling mushroom browning [136,137].

## 4. Conclusions and Future Perspective

The decline in post-harvest quality of fresh mushrooms is one of the significant challenges faced by the mushroom industry, and preservation techniques are of crucial importance in extending the shelf-life of mushrooms and enhancing their market value. This paper discusses emerging technologies in the field of mushrooms in recent years. It summarizes the applications of cold plasma treatment, electrostatic field treatment, active packaging, edible coatings, antimicrobial photodynamic therapy, electrolyzed water treatment [17], novel preservatives [18], and gene editing technology in post-harvest preservation of mushrooms, revealing their potential to improve preservation effectiveness and promote sustainable development of the industry.

Fresh mushrooms have high moisture content, delicate tissue, and high metabolic activity. During harvesting, storage, and transportation, they are susceptible to contamination and damage. Post-harvest preservation of mushrooms usually involves controlling temperature, humidity, oxygen exposure, metabolic activity, and microbial growth. Cold plasma treatment technology more efficiently inhibits microbial growth by generating active substances. Antimicrobial photodynamic therapy utilizes the recognition properties of photosensitizers to make the sterilization process more targeted. DENBA+ treatment inhibits metabolism in a milder way. Edible coatings isolate mushrooms from the external environment in a more environmentally friendly way, slowing down moisture evaporation and oxygen penetration to maintain mushroom humidity and freshness. Emerging preservation technologies better meet the requirements of green environmental protection, safety, economy, and efficient preservation, but they also have certain limitations. For instance, antimicrobial photodynamic therapy is limited by the penetration ability of light sources and cannot eliminate microorganisms hidden in the gills of edible fungi. Edible coating materials lack mechanical properties, and their stability is inferior to that of traditional film materials. Moreover, their biological preparation is complex and costly. Perhaps a composite preservation approach can be adopted, combining conventional and emerging preservation technologies, leveraging the stability and maturity of traditional techniques while harnessing the innovation and efficiency of emerging technologies to provide a viable path for developing the mushroom industry.

In the future, besides strengthening technological integration and exploring the combined application of various technologies, it is possible to monitor and control critical processes in preserving edible fungi to promote technological innovation. Furthermore, the continuous development and deepening application of new-generation information technologies are expected to propel preservation technologies in a more intelligent direction. Overall, the development of preservation technologies for edible fungi will pay more attention to quality control, energy efficiency, and environmental friendliness, contributing to the sustainable development of the industry.

## Figures and Tables

**Table 1:** Packaging film for edible fungi.

Packaging Technology	Material Property	Mushroom Species	Result	Ref.
Microperforated films	PA/PE film; 76 μm thickness; 0.5 mm hole size	*A.bisporus*	Maintained higher levels of total phenols and flavonoids; decreased the levels of relative conductivity and MDA content; downregulated specific gene expressions; reduced the browning index	2024 [74]
Microperforated films combined with high oxygen atmosphere (80% O_2_)	Polysulfone film (PSF_7000); 25 µm thickness; 25 holes; 143 µm hole size	*A. bisporus*	Maintained the desirable color; decreased MDA content; inhibited water condensation	2020 [59]
Microperforated films	PE film; 25.1 μm thickness; 8 holes; 0.3 mm hole size	*A. bisporus*	Decreased the browning index; maintained a higher concentration of 13 mushroom characteristic flavor compounds	2022 [60]
Nanocomposite packaging	Polyethylene-based packaging material loaded with nano-Ag/TiO_2_; 40 μm thickness	*A. bisporus*	Delayed the degradation of cell membrane phospholipids of mushroom; delayed the membrane lipid peroxidation process	2022 [62]
Nanocomposite packaging	Nano-Ag, nano-TiO_2_, nano-SiO_2_, nano-attapulgite, low-density polyethylene and anti-fogging agent; 40 μm thickness	*A. bisporus*	Maintained high total phenolic content and low levels of flavonoids; reduced the accumulation of melanin; delayed the browning process	2022 [63]
Nanocomposite packaging	Nano-Ag, nano-TiO_2_, nano-SiO_2_, nano attapulgite and polyethylene; 40 μm thickness	*F. filiformis*	Protected the mitochondrial integrity and function; maintained the balance of energy supplement; obtained better postharvest quality	2022 [75]
Nanocomposite packaging	Nano-Ag, nano-TiO_2_, nanoattapulgite, nano-SiO_2_ and polyethylene; 40 μm thickness	*F. filiformis*	Regulated phenylpropanoid pathway and the mitochondrial ROS production; delayed lignin deposition	2021 [76]
Nanopackaging	Nano-Ag and polyethylene; 35 μm thickness; 2.711 mg/m^3^ ozone	*A. bisporus*	Maintained a high antioxidant capacity; delayed the browning and softening processes; prolonged shelf-life up to 6~9 days	2024 [33]
Active packaging	1-MCP, molecular sieve, loaded with potassium permanganate, cinnamon essential oil microcapsule, packaging paper	*A. bisporus*	Adsorbed and removed the exogenous ethylene; delayed the softening, browning, and weight loss	2021 [66]
Active packaging	Zeolite (clinoptilolite), aҫai extract, gelatin, and glycerin	*A. bisporus*	Improved antioxidant activity; slowed down water loss and the browning process of mushroom	2021 [77]
Active packaging	Gelatin, pomegranate peel powder, and PE film	*P. ostreatus*	Inhibited the growth of bacteria; maintained firmness and color; prolonged the shelf-life up to 11 days	2020 [14]
Active packaging	MgO nanoparticles, grapeseed oil, and Poly (3-hydroxybutyrate)	*A. bisporus*	Improved antioxidant activity; inhibited the growth of bacteria; extended the shelf-life up to 6 days	2024 [67]
Intelligent packaging	Palladium on activated charcoal and 1-MCP	*A. bisporus*	Controlled 1-MCP release rate and ethylene removal rate; delayed the softening, browning, and weight loss of mushroom	2021 [72]
Intelligent packaging	Citrus pectin, cellulose nanofibers, and thymol	*A. bisporus*	Controlled adsorption/release of water and release rate of thymol; stabilized relative humidity; inhibited bacterial growth	2022 [73]

## Data Availability

No new data were created or analyzed in this study. Data sharing is not applicable to this article.
